# Cytotoxic, Antibacterial, and Antioxidant Activities of the Leaf Extract of *Sinningia bullata*

**DOI:** 10.3390/plants12040859

**Published:** 2023-02-14

**Authors:** Pin-Jui Chen, En-Shyh Lin, Hsin-Hui Su, Cheng-Yang Huang

**Affiliations:** 1 Department of Biomedical Sciences, Chung Shan Medical University, Taichung City 402, Taiwan; sean742742@gmail.com; 2 Department of Beauty Science, National Taichung University of Science and Technology, Taichung City 403, Taiwan; eslin7620@gmail.com; 3 Department of Pharmacy, Chia Nan University of Pharmacy and Science, Tainan City 717, Taiwan; indigo.ko@gmail.com; 4 Department of Medical Research, Chung Shan Medical University Hospital, Taichung City 402, Taiwan

**Keywords:** cytotoxic activities, anticancer, 4T1 mammary carcinoma, B16F10 melanoma, antioxidation, antibacterial, TPC, TFC, GC–MS analysis, *Sinningia bullata*

## Abstract

*Sinningia bullata* is a tuberous member of the flowering plant family Gesneriaceae. Prior to this work, the antibacterial, antioxidant, and cytotoxic properties of *S. bullata* were undetermined. Here, we prepared different extracts from the leaf, stem, and tuber of *S. bullata* and investigated their pharmacological activities. The leaf extract of *S. bullata*, obtained by 100% acetone (Sb-L-A), had the highest total flavonoid content, antioxidation capacity, and cytotoxic and antibacterial activities. Sb-L-A displayed a broad range of antibacterial activities against *Escherichia coli*, *Staphylococcus aureus*, and *Pseudomonas aeruginosa*. The inhibition zones of Sb-L-A ranged from 8 to 30 mm and were in the following order: *S. aureus* > *E. coli* > *P. aeruginosa*. Incubation of B16F10 melanoma cells with Sb-L-A at a concentration of 80 μg/mL caused deaths at the rate of 96%, reduced migration by 100%, suppressed proliferation and colony formation by 99%, and induced apoptosis, which was observed in 96% of the B16F10 cells. In addition, the cytotoxic activities of Sb-L-A were synergistically enhanced when coacting with the antitumor drug epothilone B. Sb-L-A was also used to determine the cytotoxic effects against 4T1 mammary carcinoma cells. Sb-L-A of 60 μg/mL boosted the distribution of the G2 phase from 1.4% to 24.4% in the B16F10 cells. Accordingly, Sb-L-A might suppress melanoma cell proliferation by inducing G2 cell-cycle arrest. The most abundant compounds in Sb-L-A were identified using gas chromatography–mass spectrometry. Overall, the collective data in this study may indicate the pharmacological potentials of Sb-L-A for possible medical applications.

## 1. Introduction

*Sinningia bullata* is a tuberous member of the flowering plant family Gesneriaceae [1]. Gesneriaceae comprises over 3400 species and have developed different strategies for overcoming water scarcity [1]. *S. bullata* is named for its bullate leaves and also produces a woolly backing to its leaves. Prior to this study, pharmacological activities of *S. bullata* were not investigated. The current study aimed to uncover cytotoxicity, antioxidation capacity, antibacterial activity, total phenolic content (TPC), and total flavonoid content (TFC) in different extracts of *S. bullata*.

Plants are a rich source of phytochemicals and secondary metabolites with numerous desired medicinal properties [2]. Phytochemicals have received attention in recent decades due to their health benefits [3]. The approach of investigating medicinal plants for therapeutic agents has proven to be a useful and productive tool for possible pharmacological applications. Some active ingredients from plant extracts have been introduced as promising anticancer drugs, such as vincristine, vinblastine, and paclitaxel [4,5,6,7,8]. One significant advantage of using natural extracts against cancer cells is their multitargeted modes of action, which provide potential synergistic behavior and polypharmacology approaches for cancer therapies. Cancer is known as one of the most impacting causes of death and morbidity globally. Cancer mortality is on the rise and has become one of the leading causes of human mortality [9,10]. Although substantial progress has been made in the control and treatment of cancer, it still caused approximately 9.9 million deaths in 2020. Many natural compounds exhibiting anticancer properties are phenols and flavonoids that can influence cell cycles [11,12,13]. In addition, pharmacological potentials and antioxidant activity are usually correlated [14,15]. Thus, TPC, TFC, and antioxidation activity of different extracts are worth determining to evaluate the pharmacological potentials of *S. bullata*.

Antimicrobial drug resistance is an increasing threat to global public health [16]. The cases of antibiotic-resistant bacterial infections are alarmingly increasing [17,18]. Multidrug-resistant pathogenic bacteria are spreading rapidly worldwide and can become untreatable. For example, *Staphylococcus aureus* exhibits a remarkable ability to develop antibiotic resistance and causes approximately 19,000 deaths per year in the United States [19]. Accordingly, the continuous development of clinically useful small-molecule antibiotics is greatly needed to target these bacteria and treat antibiotic-resistant infections. Many kinds of natural extracts from plants have antimicrobial activities and are being used as alternatives [20]. Thus, investigating the antibacterial activity of different *S. bullata* extracts is of considerable interest for drug development.

**Figure 1:**
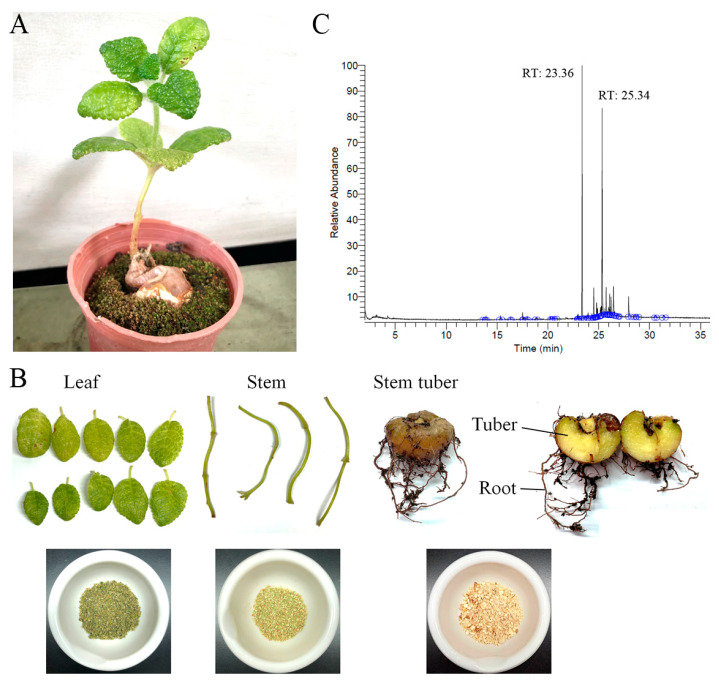
Preparation of different extracts from *S. bullata*. (**A**) *S. bullata* is a tuberous member of the flowering plant family *Gesneriaceae*. *S. bullata* has bullate leaves. (**B**) Various parts of *S. bullata*, i.e., the leaf, stem, and stem tuber, were collected, dried, cut into small pieces, pulverized into powder, and extracted using water, methanol, ethanol, and acetone to assess whether these extracts have pharmacological potentials. The roots were removed from the tuber and were not involved in this study. (**C**) GC–MS analysis of the leaf extract obtained by 100% acetone. GC chromatogram of compounds in this extract is shown. Compounds were identified by matching generated spectra with NIST 2011 and Wiley 10th edition mass spectral libraries.

In this study, various parts of *S. bullata*, i.e., the leaf, stem, and tuber, were collected, dried, cut into small pieces, pulverized into powder, and extracted using water, methanol, ethanol, and acetone to assess whether these extracts have pharmacological potentials (Figure 1). Among these extracts, the leaf extract of *S. bullata* obtained by 100% acetone (Sb-L-A) exhibited the highest cytotoxic activities against B16F10 melanoma and 4T1 mammary carcinoma cells. The cytotoxicity activity of Sb-L-A combined with the anticancer drug epothilone B (EpoB) against the melanoma cells was also studied. The chemical composition of Sb-L-A was analyzed via gas chromatography–mass spectrometry (GC–MS). Further studies should directly focus on determining whether and how this extract can be used as an alternative medicine.

## 2. Results

This study aimed to determine the pharmacological potentials of *S. bullata* (Figure 1) for possible medical applications. Various parts of *S. bullata*, i.e., the leaf (Sb-L), stem (Sb-S), and tuber (Sb-T), were collected, dried, cut into small pieces, pulverized into powder, and extracted using water (W), methanol (M), ethanol (E), and acetone (A) to assess whether these extracts have the antibacterial, antioxidant, and cytotoxic activities.

### 2.1. Total Phenolic Content (TPC)

Prior to this study, the TPC of *S. bullata* remained undetermined. Given that many polyphenols can be developed as drug candidates from the active confirmation of in vitro screens or in vivo evaluations [21,22], it is worth analyzing the TPC of *S. bullata* extracts. TPC was quantified using the modified Folin–Ciocalteu method. TPC values ranged from 6.5 mg GAE/g for Sb-T-W (the tuber extract obtained using 100% water) to 49.7 mg GAE/g for Sb-S-A (the stem extract obtained using 100% acetone). Overall, the stem was the major source of phenols in *S. bullata* and acetone was the most effective extracting solvent for the TPC (Table 1).

TPC was quantified using the modified Folin–Ciocalteu method. The absorbance of blue color developed was measured at 750 nm by using a UV/VIS spectrophotometer. The results were compared with the standard curves of gallic acid (GAE) and were expressed as mg equivalent/g dry weight (mg GAE/g).

### 2.2. Total Flavonoid Content (TFC)

Flavonoids are natural products with several structure-dependent biological and pharmacological activities [23,24,25]. Thus, the TFC of *S. bullata* was also determined (Table 2). TFC was quantified using the aluminum chloride colorimetric method. TFC values ranged from 16.0 mg QUE/g for Sb-T-W to 65.8 mg QUE/g for Sb-L-A. Accordingly, the leaf was the major source of flavonoids in *S. bullata* (Table 2).

TFC was quantified using the aluminum chloride calorimetric method. The absorbance of extracts and standard solutions was measured at 510 nm. The results were expressed as mg of QUE equivalent/g dry weight (mg QUE/g).

### 2.3. Antioxidant Activity

**Figure 2:**
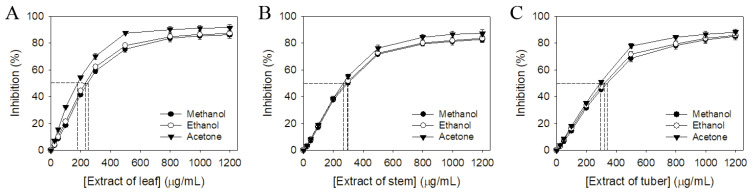
Antioxidant activity of different extracts of *S. bullata*. The antioxidant activities of extracts from (**A**) leaf, (**B**) stem, and (**C**) tuber prepared using methanol, ethanol, and acetone were evaluated by DPPH radical scavenging assay.

Pharmacological potentials and antioxidant activity are usually correlated [14,15]. Thus, we evaluated the antioxidant activity of different extracts of *S. bullata* using 1,1-diphenyl-2-picrylhydrazyl (DPPH) radical scavenging assay (Figure 2). DPPH assay is the most common method to assess the antioxidant capacity of plants. The antioxidant capacities of different *S. bullata* extracts were quantified by IC_50_ values, calculated from the DPPH titration curves (Figure 2 and Table 3). IC_50_ values of water extracts of *S. bullata* were too low to determine. As compared to their IC_50_ values, the antioxidant capacity of extracts of *S. bullata* followed the order: leaf > stem > tuber. Sb-L-A showed the highest antioxidant capacity with an IC_50_ value of 180.8 ± 2.8 μg/mL.

IC_50_ values were calculated from the titration curves of the DPPH assay by determining the concentration of the extract needed to achieve the midpoint value for inhibition. IC_50_ values of water extracts of *S. bullata* were too low to determine.

### 2.4. Antibacterial Activity

The antibacterial activities of different *S. bullata* extracts were investigated using the agar well diffusion method. The antibacterial capacities were quantified by the zone of inhibition (Table 4). Human pathogens (*Escherichia coli*, *Staphylococcus aureus*, and *Pseudomonas aeruginosa*) were used for this analysis. The extracts showed differences in antibacterial activities with the zone of inhibition. The inhibition zones of Sb-L-A ranged from 8 to 30 mm. The water extracts of *S. bullata* did not inhibit the growth of these bacteria. Tuber extracts of *S. bullata* did not inhibit the growth of the Gram-negative *E. coli* and *P. aeruginosa*. Sb-L-A showed the highest antibacterial activities against these three bacteria. Overall, the antibacterial activities of Sb-L-A followed the order: *S. aureus* > *E. coli* > *P. aeruginosa*.

### 2.5. Anticancer Potential

**Figure 3:**
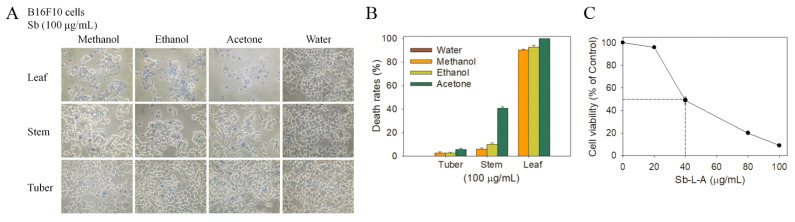
The cytotoxic effect of different *S. bullata* extracts against B16F10 cells. (**A**) Trypan blue dye exclusion staining. The cytotoxic effects of different *S. bullata* extracts against B16F10 cells were estimated with trypan blue assay after 24 h of incubation. The B16F10 cells incubated with Sb-L-A of 100 μg/mL were almost dead. (**B**) The death rates of B16F10 cells. The anti-B16F10 activity of *S. bullata* extracts followed the order: leaf > stem > tuber. (**C**) MTT cell viability assay. Sb-L-A (20, 40, 80, and 100 μg/mL) were added to cell culture medium in a 96-well plate with B16F10 cells (3 × 10^3^). Sb-L-A was found to significantly inhibit B16F10 cells growth in a dose dependent manner with IC_50_ value of 39.8 ± 1.6 μg/mL.

Prior to this study, very little was known about whether extracts of *Sinningia* plants can suppress the growth of cancer cells. Malignant melanoma is the most dangerous and the most common type of skin cancer [26]. Considering that many natural products exhibit anticancer properties towards skin cancers, the highly metastatic B16F10 melanoma cells were selected for this investigation. The monolayers prepared in 96-well microtitration plates for B16F10 cells were inoculated with *S. bullata* extracts at concentrations of 100 μg/mL per well (Figure 3A). The cytotoxic effect of *S. bullata* extracts was estimated with a trypan blue assay after 0 and 24 h of incubation, and the anti-B16F10 activity of *S. bullata* extracts followed the order: leaf > stem > tuber. The B16F10 cells incubated with Sb-L-A of 100 μg/mL were almost dead (Figure 3B). The water extracts of *S. bullata* did not cause any cytotoxic effect on the survival of B16F10 cells. We also examined the cytotoxic effect using 3-(4,5-dimethylthiazol-2-yl)-2,5-diphenyltetrazolium bromide (MTT). Through MTT assay (Figure 3C), Sb-L-A was found to significantly inhibit B16F10 cells growth in a dose dependent manner with IC_50_ value of 39.8 ± 1.6 μg/mL. Consequently, these results indicated the anticancer potential of Sb-L-A.

### 2.6. Cytotoxic Activities against B16F10 Melanoma Cells

**Figure 4:**
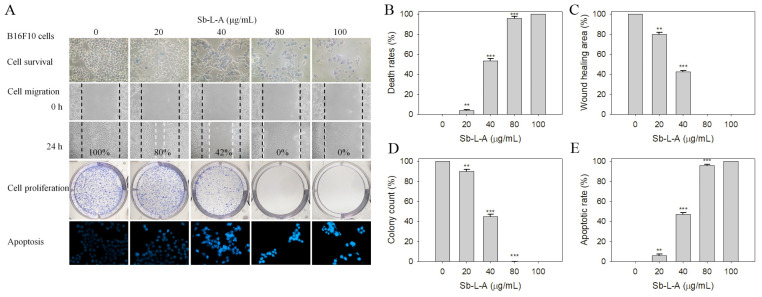
The cytotoxic effects of Sb-L-A against B16F10 cells. (**A**) Effects on cell survival, migration and proliferation, and apoptosis. (**B**) Trypan blue dye exclusion staining results. B16F10 cells incubated with Sb-L-A at concentrations of 20, 40, 80, and 100 μg/mL. (**C**) The wound healing assay. Sb-L-A significantly inhibited cell migration. (**D**) Clonogenic formation assay results. Pretreatment with Sb-L-A strongly suppressed the proliferation and colony formation of B16F10 cells. (**E**) Hoechst staining results. Apoptosis induced by Sb-L-A with DNA fragmentation was observed in B16F10 cells. ** *p* < 0.01 and *** *p* < 0.001 compared with the control group.

We found that Sb-L-A exhibited cytotoxicity on melanoma cell survival, migration, and proliferation and also induced cell apoptosis (Figure 4A). The death rate of B16F10 cells caused by Sb-L-A was estimated with trypan blue staining assay (Figure 4B). Incubation with Sb-L-A at concentrations of 20, 40, 80, and 100 μg/mL caused the deaths of B16F10 cells at the rates of 4%, 53%, 96%, and 100%, respectively. The wound-healing assay, typically used to study cell migration and cell–cell interaction, showed that Sb-L-A strongly reduced the migration of B16F10 cells. After 24 h of incubation, Sb-L-A of 80 μg/mL completely inhibited B16F10 cell migration (Figure 4C). The clonogenic formation assay (Figure 4D), based on the ability of a single cell to grow into a colony, revealed that pretreatment with Sb-L-A of 80 μg/mL significantly suppressed the proliferation and colony formation of B16F10 cells (99%). Hoechst staining, widely used to distinguish the compact chromatin of apoptotic nuclei, showed Sb-L-A (80 μg/mL)-induced apoptosis with DNA fragmentation in 96% of the B16F10 cells. Accordingly, Sb-L-A exhibited cytotoxic activities against B16F10 melanoma cells.

### 2.7. Cytotoxic Activities against 4T1 Mammary Carcinoma Cells

**Figure 5:**
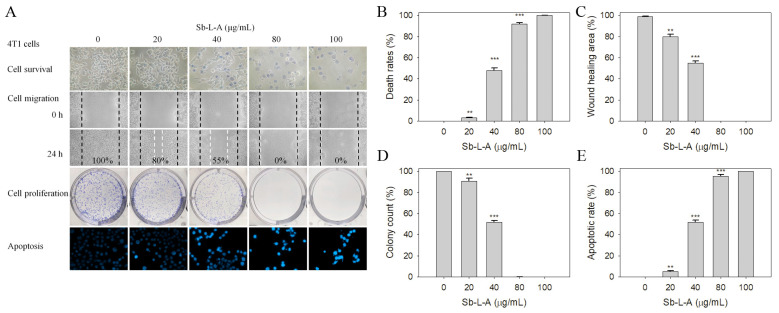
The cytotoxic effects of Sb-L-A against 4T1 cells. (**A**) Effects on cell survival, migration and proliferation, and apoptosis. (**B**) Trypan blue dye exclusion staining results. 4T1 cells incubated with Sb-L-A at concentrations of 20, 40, 80, and 100 μg/mL. (**C**) The wound healing assay. Sb-L-A significantly inhibited cell migration. (**D**) Clonogenic formation assay results. Pretreatment with Sb-L-A strongly suppressed the proliferation and colony formation of 4T1 cells. (**E**) Hoechst staining results. Apoptosis induced by Sb-L-A with DNA fragmentation was observed in 4T1 cells. ** *p* < 0.01 and *** *p* < 0.001 compared with the control group.

The 4T1 mammary carcinoma is a transplantable breast cancer cell line that is highly tumorigenic and invasive [27,28]. The 4T1 cells can spontaneously metastasize from the primary tumor in the mammary gland to multiple distant sites, including the lymph nodes, blood, liver, lung, brain, and bone. Sb-L-A was also used to determine the cytotoxic effects against 4T1 cells (Figure 5A). Incubation of 4T1 cells with Sb-L-A at a concentration of 80 μg/mL caused deaths at the rate of 92% (Figure 5B), reduced migration by 100% (Figure 5C), suppressed proliferation and colony formation by 99% (Figure 5D), and induced apoptosis, which was observed in 95% of the 4T1 cells (Figure 5E). Accordingly, Sb-L-A also exhibited significant cytotoxic activities against 4T1 mammary carcinoma cells.

### 2.8. Gas Chromatography–Mass Spectrometry (GC–MS) Analysis of Sb-L-A

Given that Sb-L-A had the highest cytotoxic activities (Figure 3), antioxidation capacity (Figure 2 and Table 3), and antibacterial activities (Table 4), the most abundant compounds in this extract were determined using GC–MS. The GC chromatogram (Figure 1C) showed compounds detected in Sb-L-A. These compounds were identified by matching generated spectra with NIST 2011 and Wiley 10th edition mass spectral libraries. Contents tentatively identified in this extract were γ-tocopherol, β-sitosterol, stigmasterol, fuscumol, β-ionone, α-tocopherol, neophytadiene, 7,11-hexadecadienal, phytanol, stigmasta-3,5-diene, 2,4-di-tert-butylphenol, 3-(3,7-dimethyl-octa-2,6-dienyl)-4-hydroxy-benzaldehyde, octadecanoic acid, 3,7,11-trimethyl-1-dodecanol, cis-jasmonolactone, 6-[(2Z)-2-butenyl]-1,5,5-trimethyl-1-cyclohexene, δ-tocopherol, and phytol.

### 2.9. Sb-L-A Suppressed Melanoma Cell Proliferation by Inducing G2 Cell-Cycle Arrest

**Figure 6:**
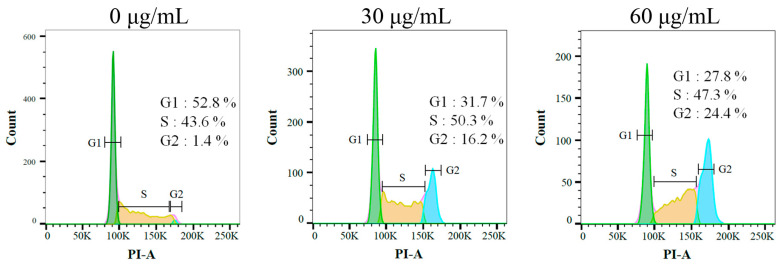
Flow analysis results. B16F10 cells were treated with Sb-L-A at indicated concentrations for 24 h and fixed with 70% alcohol overnight. The cell suspension was stained with propidium iodide (PI) for 30 min and subjected to flow cytometry.

The effect of Sb-L-A against the cell-cycle progression of B16F10 cells was examined by using flow cytometry (Figure 6). The melanoma cells were treated with Sb-L-A at concentrations of 30 and 60 μg/mL. Incubation with Sb-L-A of 30 and 60 μg/mL boosted the distribution of the G2 phase from 1.4% to 16.2% and 24.4% in the B16F10 cells, respectively. Accordingly, Sb-L-A might suppress melanoma cell proliferation by inducing G2 cell-cycle arrest.

### 2.10. Co-Treatment of Sb-L-A with Epothilone B against B16F10 Cells

**Figure 7:**
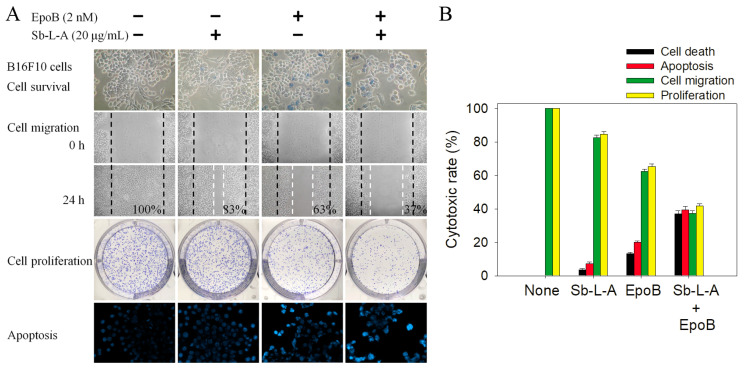
The potential synergistic cytotoxic effects of EpoB with Sb-L-A. (**A**) Incubation of B16F10 cells with EpoB and Sb-L-A. Sb-L-A (20 μg/mL) and EpoB (2 nM) were co-used to investigate the cytotoxic effects on cell survival, migration, proliferation, and apoptosis. (**B**) The collective data suggested potential synergistic cytotoxic effects because the co-treatment of Sb-L-A with EpoB could cause more deaths of the cancer cells, further inhibit the migration and proliferation, and produce more DNA fragmentations in B16F10 cells.

We also evaluated the cooperative effect of Sb-L-A with epothilone B (EpoB) against the melanoma cells (Figure 7). EpoB is the antitumor drug widely used for the treatment of ovarian cancer, lung cancer, brain cancer, breast cancer, and gastric cancer [29]. EpoB is a stabilizing tubulin antagonist and, therefore, inhibits microtubule function [29,30]. Given that microtubules are essential to cell division, EpoB acts to stop cells from properly dividing. Sb-L-A of 20 μg/mL, capable of inducing a minor cytotoxic effect, was selected for this co-treatment experiment (Figure 7A). Incubation of B16F10 cells with Sb-L-A, EpoB (2 nM), and co-treatment of Sb-L-A with EpoB caused deaths at the rate of 3%, 13%, and 37%, reduced migration by 18%, 38%, and 63%, suppressed proliferation and colony formation by 16%, 35%, and 59%, and induced apoptosis observed in 7%, 20%, and 40% of the B16F10 cells, respectively (Figure 7B). These results indicated potential synergistic cytotoxic effects because the co-treatment of Sb-L-A with EpoB could cause more deaths of the cancer cells, further inhibit the migration and proliferation, and produce more DNA fragmentations in B16F10 cells. The anticancer drug 5-fluorouracil was also used with Sb-L-A for investigating the possible synergistic effect; however, no additional effect was found (data not shown). Given the co-cytotoxic activities, the antitumor drug EpoB might be co-used with Sb-L-A for better anticancer applications. However, this speculation must be further demonstrated experimentally and clinically.

## 3. Discussion

Plant-derived natural products, whether as pure compounds or standardized extracts, have received attention due to their many health benefits and offer countless prospects for new drug discovery [31,32,33]. Natural products possess structural complexity, diversity, and chirality with attractive functions and biological activities that have significantly impacted drug development initiatives [34]. Plant-derived herbs and drugs have been traditionally used as antitumor agents for many centuries and are increasingly used in modern societies [35]. Many natural products with biological activities are alkaloids, flavonoids, tannins, terpenoids, saponins, and phenolic compounds. These bioactive compounds in plants can be effective for humans in treating various disorders due to their antioxidant, anti-inflammatory, antibacterial, and anticancer activities [36]. Thus, it is still worth determining the pharmacological potentials for the uninvestigated plants, such as extracts of *S. bullata,* used in this study (Figure 1). We analyzed TPC (Table 1), TFC (Table 2), antibacterial activities (Table 4), antioxidant capacity (Figure 2 and Table 3), and the cytotoxicity of different extracts from *S. bullata*. Further research can directly focus on determining whether *S. bullata* extracts could be a potential natural alternative or complementary therapy, such as to be an adjuvant or ointment for the treatment of melanoma cancer.

Pharmacological potentials and antioxidant activity are usually correlated [14,15]. Sb-L-A showed the highest DPPH radical scavenging activity with an IC_50_ value of 180.8 ± 2.8 μg/mL, while the tuber extracts had poor radical scavenging potential (Table 3). Accordingly, Sb-L-A was chosen for the GC–MS analysis to determine the active ingredients. Sb-L-A also had the highest TFC and cytotoxic and antibacterial activities. γ-Tocopherol, δ-tocopherol, and α-tocopherol were found in Sb-L-A. The antioxidant functions of these tocopherols (vitamin E) have been well confirmed in vivo and in vitro [37]. β-Ionone in Sb-L-A is also known to possess high antioxidant activities [38]. The antioxidation capacity of Sb-L-A might be from the co-effects of these compounds.

Cancer is one of the leading causes of human mortality [9,10]. Cancer cells’ traits, such as sustained proliferation, resistance to cell death, angiogenesis capacity, invasion, evasion of immune surveillance, and metastasis, could be the targets of the bioactive compounds of plants [39]. In this study, we examined the cytotoxic effects of Sb-L-A on the survival, apoptosis, proliferation, and migration of 4T1 (Figure 5) and B16F10 cells (Figure 4). Cell metastasis is a complicated process, which gradually leads to cancer propagation. The cancer cells are forced to lose epithelial-like features and invade the body through blood vessels, spreading into distant organs. In addition, this process is usually associated with drug resistance and disease recurrence [39]. Conventional cancer treatments commonly involve radiotherapy and chemotherapy but with several adverse effects and other critical disorders. Recently, natural products as potential anticancer agents have also been used and studied in many cancer models [35,40]. One significant advantage of using natural extracts against cancer cells is their multitargeted modes of action, which provide potential synergistic behavior and polypharmacology approaches for cancer therapies. Currently, promising plant-based anticancer medicines such as vincristine, vinblastine, and paclitaxel have been developed and used in clinical applications [4,5]. β-Ionone identified in Sb-L-A is also known to have significant antiproliferative, antimetastatic, and apoptosis-induction activities, both in vitro and in vivo [41]. Given that the combination of Sb-L-A (20 μg/mL) with the anticancer drug EpoB (2 nM) could synergistically enhance the cytotoxicity against the B16F10 cells (Figure 7), how Sb-L-A can co-act with EpoB to improve the chemosensitivity of EpoB should be further elucidated.

Cancer progression is associated with the dysfunction of checkpoint controls, which regulate normal passage through the cell cycle [42]. The G2 cell-cycle checkpoint is a critical genome guardian of tumor cells and, therefore, G2 checkpoint abrogation has been considered to be a promising therapeutic anticancer target, such as several cell cycle proteins [42]. The flow cytometry results indicated that Sb-L-A could promote the distribution of the G2 phase and decreased the cell proportion in the G1 and S phases in B16F10 melanoma cells (Figure 6). Accordingly, Sb-L-A might suppress melanoma cell proliferation by inducing G2 cell-cycle arrest. Currently, we are investigating the cellular signaling pathways that trigger this G2 arrest in B16F10 melanoma cells.

There is widespread consensus that antimicrobial resistance represents an emerging and alarming threat to human health worldwide [17,18]. As recognized by the Infectious Diseases Society of America, ESKAPE pathogens (*Enterococcus faecium*, *S. aureus*, *Klebsiella pneumoniae*, *Acinetobacter baumanii*, *P. aeruginosa*, and *Enterobacter* species) cause the majority of US hospital infections and effectively “escape” the effects of antibacterial drugs [16]. With the incidence of antimicrobial resistance rising globally, there is a continuous need for development of new antimicrobial molecules. In this study, we found that Sb-L-A inhibited the growth of both the Gram-positive and Gram-negative bacteria (Table 4). The antibacterial activities of Sb-L-A followed the order: *S. aureus* > *E. coli* > *P. aeruginosa*. *S. aureus* exhibits a remarkable ability to develop antibiotic resistance and causes approximately 19,000 deaths per year in the United States [19]. Accordingly, the continuous drug development is greatly needed to target *S. aureus* and treat drug-resistant infections.

Besides *S. bullata*, some other Sinningia species also have cytotoxic, antibacterial, and antioxidant activities. There are two scientific articles, clearly indicating that extracts of *S. allagophylla* [43] and *S. mauroana* [44] have strong cytotoxic activities. Compounds isolated from *S. aggregata* [45], *S. reitzii* [46], and *S. leucotricha* [47] have been further analyzed in cytotoxic activities. The antibacterial and antioxidant properties of extracts and the isolated compounds from *S. hatschbachii* [48], *S. magnifica* [49], *S. aggregata* [50], *S. warmingii* [51], *S. mauroana* [44,52], and *S. reitzii* [53] have also been investigated experimentally. Unlike results in this study, however, most of these extracts did not have significant antibacterial activities. Whether these significant disparities are due to inherent differences among the species, different growth conditions of the plants (the soil, fertilizer, altitude, and so on), additional plant–environment interactions, the use of different assay methods, the use of different solvent for extractions, and/or the effect of different investigators remains unknown. We noticed that the focusing studied targets of these Sinningia plants are different. Although we prepared different extracts from various parts of *S. bullata*, i.e., the leaf, stem, and tuber, we only found that the leaf extracts obtained using acetone had the highest cytotoxic activities, antioxidation capacity, and antibacterial activity against the Gram-positive *S. aureus*. Similarly, as in most studies mentioned above, tuber extracts of *S. bullata* did not show any antibacterial activity (with the inhibition zone of 0) (Table 4). Furthermore, even using acetone as the extraction solvent, tuber extracts of *S. bullata* also had very low cytotoxic activities as compared to the leaf extract (Figure 3). Accordingly, the most abundant compounds in this extract were tentatively deduced using GC–MS. We also noted that some compounds contained in tubers of Sinningia species, mainly analyzed by using nuclear magnetic resonance spectroscopy, such as aggregatin E [45], aggregatin F [45], leucotrichoic acid [47], 6-hydroxy-7-methoxy-2-O-methylduniol [48], 5,6-dihydroxy-7-methoxy-α-dunnione [53], 8-hydroxy-6,7-dimethoxy-α-dunnione [53], warmingiin A [51], and warmingiin B [51], were not found in our study. Although GC–MS is widely used in the analysis of chemical compositions in plant extract and provides enhanced sample identification, higher sensitivity, and an increased range of analyzable samples in a reasonable time, GC–MS is not capable of directly analyzing compounds in plant extract that are nonvolatile, polar, or thermally labile. It is possible that the major compounds in the acetone extract of *S. bullata* leaf have not been detected via GC–MS. Given that some plant medicines are only produced in some specific species, for example, for artemisinin that is abundant in *Artemisia annua*, but not in *Artemisia apiacea*, more studies should be needed to determine the chemical compositions of different parts of these different *Sinningia* plants for further possible medical applications. To understand the cytotoxic mechanisms of the acetone extract, our laboratory is currently attempting to determine the molecular target(s) in the Gram-positive *S. aureus* and the melanoma cell.

In conclusion, we evaluated the TPC, TFC, cytotoxicity, antibacterial activities, and antioxidant capacity of different parts of *S. bullata* extracts by using methanol, ethanol, acetone, and distilled water. The cytotoxic effects of Sb-L-A on the survival, apoptosis, proliferation, and migration of B16F10 and 4T1 cancer cells were examined. The abundant ingredients in this extract were determined via GC–MS to obtain a better understanding of which compound(s) may be active, alone or in combination, in these biological activities. These results might indicate the pharmacological potentials of *S. bullata* for further clinical anticancer chemotherapies.

## 4. Materials and Methods

### 4.1. Chemicals and Cell Culture

All chemicals were purchased from Sigma-Aldrich (St. Louis, MO, USA) and were of analytical grade. The 4T1 mammary carcinoma [54,55] and B16F10 murine melanoma [56,57,58] cell lines were obtained from the Food Industry Research and Development Institute, Hsinchu, Taiwan. Cells were cultured in Dulbecco’s modified Eagle’s medium (DMEM) and incubated at 37 °C in a humidified incubator with 5% CO_2_. Medium was supplemented with 10% fetal bovine serum (FBS), 100 unit/mL penicillin, 100 μg/mL streptomycin, and 4 mM L-glutamine.

### 4.2. Plant Materials and Extract Preparations

*S. bullata* was collected south of Mount Babo Hagai (about an elevation of 400 m), Puli, Nantou County, Taiwan, in December 2020. This plant was identified by Dr. Zhong-Bao Zhang, the expert at Guoguang Flower Market and Taiwan Provincial Flower Marketing Cooperative, Taiwan. Leaves, stems, and tubers of *S. bullata* were collected, dried, cut into small pieces, and pulverized into powder (Figure 1). Extractions were carried out by placing 1 g of plant powder into 250 mL conical flask. To the flask, 100 mL of 100% methanol, ethanol, acetone, or distilled water were added; the flask was shaken on an orbital shaker for 5 h. The resultant extract was filtered using a 0.45 μm filter and stored at −80 °C until use. Solvents (water, methanol, ethanol, and acetone) in extracts were removed using hot air circulation oven at 40 °C. Approximately 252 mg of Sb-T-M, 240 mg of Sb-S-M, 304 mg of Sb-L-M, 176 mg of Sb-T-E, 224 mg of Sb-S-E, 288 mg of Sb-L-E, 128 mg of Sb-T-A, 196 mg of Sb-S-A, 324 mg of Sb-L-A, 184 mg of Sb-T-W, 272 mg of Sb-S-W, and 290 mg of Sb-L-W were obtained from 1000 mg of dried plant powder. For all assays in this study, the extract powder was dissolved in 20% DMSO to make a stock solution at a concentration of 20 mg/mL. For the MTT and anticancer cell assays, the stock was diluted with the supplemented culture medium to the indicated assay concentrations. The cancer cells were incubated with the resulting extract solutions or the culture medium with 0.1% DMSO, referenced as the treatment or control group. For the antioxidant and antibacterial assays, the stock was diluted with 10% DMSO to the indicated assay concentrations.

### 4.3. Determination of TPC

The quantification of TPC was carried out using the modified Folin–Ciocalteu method [59]. The absorbance of the blue color developed was measured at 750 nm by using a UV/VIS spectrophotometer (Hitachi U 3300, Hitachi High-Technologies, Tokyo, Japan) [58]. GAE of different concentrations was used as the positive control, and the results of *S. bullata* extracts were compared with the standard curves of GAE and are expressed as mg equivalent/g dry weight of plant. Values show mean standard deviation of at least three independent experiments.

### 4.4. Determination of TFC

The quantification of TFC was carried out using the aluminum chloride colorimetric method [60]. The absorbance of extracts and standard solutions was measured at 510 nm by using a UV/VIS spectrophotometer (Hitachi U 3300, Hitachi High-Technologies, Tokyo, Japan) [58]. QUE of different concentrations was used as the positive control, and the results of *S. bullata* extracts were compared with the standard curves of QUE and are expressed as mg of QUE equivalent/g dry weight of plant. Values show mean standard deviation of at least three independent experiments.

### 4.5. Determination of Antioxidant Activity by DPPH Radical Scavenging Assay

The antioxidant potential of the plant extracts was determined using a DPPH assay [61]. DPPH was dissolved in absolute ethanol. Briefly, 180 μL of DPPH working solution was mixed with 20 μL of extracts in a 96-well plate and was incubated in the dark at RT for 15 min, and absorbance was measured using a microplate reader at 517 nm. L-Ascorbic acid dissolved in 10% DMSO was used as the positive control, with IC_50_ of 15.9 μg/mL. Values show mean standard deviation of at least three independent experiments. DPPH free radical scavenging activity was determined using the formula:

%Radical scavenging activity = (Control OD − Sample OD)/Control OD × 100. The absorbance was measured at 517 nm.

### 4.6. GC-MS Analysis

Phytochemical components of Sb-L-A were determined by GC-MS. The filtered sample was analyzed using Thermo Scientific TRACE 1300 Gas Chromatograph with a Thermo Scientific ISQ Single Quadrupole Mass Spectrometer system (Waltham, MA, USA) [54,56,57,58]. The column used was Rxi-5ms (30 m × 0.25 mm i.d. × 0.25 μm film). Helium was used as the carrier gas at a constant flow rate of 1 mL/min. The initial oven temperature was 40 °C and it was maintained at this temperature for 3 min; the temperature was gradually increased to 300 °C at a rate of 10 °C/min and was maintained for 1 min. The temperature of the injection port was 300 °C and the flow rate of helium was 1 mL/min. The compounds discharged from the column were detected using a quadrupole mass detector. The ions were generated using electron ionization method. The temperatures of the MS quadrupole and source were 150 and 300 °C, respectively; electron energy was 70 eV; the temperature of the detector was 300 °C; the emission current multiplier voltage was 1624 V; the interface temperature was 300 °C; and the mass range was from 29 to 650 amu. The relative mass fraction of each chemical component was determined by the peak area normalization method. Compounds were identified by matching generated spectra with NIST 2011 and Wiley 10th edition mass spectral libraries.

### 4.7. Trypan Blue Cytotoxicity Assay

The trypan blue cytotoxicity assay was performed to assess cell death [62]. The cancer cells (1 × 10^4^) were incubated with different extracts in a 100 μL volume. After 24 h, the cytotoxic potentiality exhibited by the extract was estimated by performing a trypan blue cytotoxicity assay.

### 4.8. Chromatin Condensation Assay

The apoptosis in cancer cells was assayed with Hoechst 33342 staining [63]. The cells were seeded in 96-well plates at a density of 5 × 10^3^ cells per well in a volume of 200 μL of culture medium. Cells were allowed to adhere for 16 h. After different treatments, cells were incubated for an additional 24 h, washed with PBS, and stained with Hoechst dye (1 μg/mL) in the dark at RT for 10 min. Cells were imaged using the ImageXpress Pico (Molecular Devices, San Jose, CA, USA). Image acquisition was performed on each well using a 20x magnification and a 6 × 6 square image scan on the DAPI filter cubes. Image analyses were performed on the images obtained from the ImageXpress Pico instrument (Molecular Devices, CA, USA) using the CellReporterXpress Version 2 software. The apoptotic index was calculated as follows:

apoptotic index = apoptotic cell number/(apoptotic cell number + nonapoptotic cell number).

### 4.9. Clonogenic Formation Assay

A clonogenic formation assay [64] was used to assess the cell growth to study whether Sb-L-A could inhibit cell proliferation. Briefly, the cells were seeded on 6-well plates at a density of 1 × 10^3^ cells per well. After different treatments, plates were incubated for 5–7 days to allow clonogenic growth. After washing with PBS, colonies were fixed with methanol and stained with 0.5% crystal violet for 20 min, and the number of colonies was counted under a light microscope.

### 4.10. Wound-Healing Assay

An in vitro migration (wound healing) assay was performed as described previously [65]. Briefly, the cancer cells were seeded in 24-well plates, incubated in serum-reduced medium for 6 h, wounded in a line across the well with a 200 μL pipette tip, and washed twice with the serum-reduced medium. After different treatments, cells were incubated for 24 h to allow migration.

### 4.11. Antibacterial Activities

The antibacterial activities of different extracts from *S. bullata* were analyzed using an agar well diffusion assay [66]. Colonies of bacteria (*S. aureus*, *P. aeruginosa*, and *E. coli*) were diluted to prepare a 0.1 McFarland standard suspension. Then, the bacteria were inoculated into sterile Petri dishes of 60 mL of Muller–Hinton agar plates. The plates were shaken gently to allow even mixing of bacterial cells and agar. All samples were dissolved in 30% DMSO to furnish 22 mg/mL. Exactly 90 μL of each extracted sample (6.0 mm diameter disc) was transferred onto the plate and incubated at 37 °C for 12 h. The diameters of the inhibition zones were recorded. The inhibition zone is an indication of the antibacterial activity, which increases in size as the potency of the extract increases. Ampicillin and 10% DMSO were used as the positive and negative controls, respectively. For *E. coli*, *S. aureus*, and *P. aeruginosa*, the inhibition zones of ampicillin at concentration of 1 mg/mL were 14 ± 1, 26 ± 2, and 9 ± 1 mm, respectively. Values show mean standard deviation of at least three independent experiments.

### 4.12. Flow Analysis

Cell cycle analysis was performed via flow cytometry [56]. B16F10 cells were treated with DMSO or Sb-L-A for 24 h and harvested with trypsin. Harvested cells were washed, resuspended in PBS with 1% FBS, and fixed with cold ethanol (70%). Fixed cells were washed, incubated in PBS buffer for 5 min, and resuspended in PI/RNase solution (PBS, RNase, and 50 μg/mL PI) for staining. The resultant cells were stained for 30 min at 37 °C in the dark and analyzed via flow cytometry with a BD FACSCanto II system (BD Biosciences, San Jose, CA, USA). The distribution of each phase was calculated and visualized directly via FlowJo v10 software (Tree Star, Inc., Ashland, OR, USA).

### 4.13. MTT Cell Viability Assay

The effect of Sb-L-A on the viability of B16F10 cells was evaluated by MTT assay [67]. Initially, serial dilutions of Sb-L-A (20, 40, 80, and 100 μg/mL) were added to cell culture medium in a 96-well plate with B16F10 cells (3 × 10^3^) per well to a final volume of 100 μL. After 24 h of incubation, 30 μL of an MTT solution (5 mg/mL MTT in PBS) were added to each well. The treated cells were further incubated with MTT solution for 4 h at 37 °C in the dark. The formed formazan crystals were dissolved in 100 μL of DMSO at 37 °C for 10 min. The data was measured on a plate spectrophotometer at 540 nm. Assays were performed in triplicate. EpoB (0, 1, 2, 5, 10, 15 nM) and the culture medium with 0.1% DMSO were used as the positive and negative controls, respectively. IC_50_ of EpoB was 7.54 nM. Values show mean standard deviation of at least three independent experiments.

## Figures and Tables

**Table 1:** TPC of *S. bullata* extracts.

	Leaf	Stem	Tuber
Water	16.4 ± 0.4	16.6 ± 0.3	6.5 ± 0.2
Methanol	39.6 ± 1.0	46.6 ± 0.8	21.7 ± 0.4
Ethanol	40.3 ± 0.9	47.1 ± 1.1	23.7 ± 0.3
Acetone	46.2 ± 1.2	49.7 ± 0.8	28.4 ± 0.8

**Table 2:** TFC of *S. bullata* extracts.

	Leaf	Stem	Tuber
Water	31.4 ± 0.4	21.1 ± 0.3	16.0 ± 0.3
Methanol	62.1 ± 1.2	50.3 ± 0.8	31.9 ± 0.7
Ethanol	63.0 ± 1.0	51.9 ± 0.6	33.8 ± 0.6
Acetone	65.8 ± 1.3	52.5 ± 0.6	37.1 ± 0.7

**Table 3:** Antioxidant activities of *S. bullata* extracts.

	IC_50_ (μg/mL)		
Solvent	Leaf	Stem	Tuber
Methanol	250.9 ± 4.2	298.4 ± 4.6	340.1 ± 5.6
Ethanol	232.2 ± 4.0	294.0 ± 5.2	321.2 ± 4.2
Acetone	180.8 ± 2.8	266.3 ± 3.8	293.2 ± 4.6

**Table 4:** Inhibition zone of *S. bullata* extracts.

		Zone of Inhibition (mm)		
Material	Solvent	*E. coli*	*S. aureus*	*P. aeruginosa*
Leaf	Water	0 ± 0	0 ± 0	0 ± 0
	Methanol	10 ± 1	27 ± 1	7 ± 0
	Ethanol	10 ± 0	27 ± 1	8 ± 0
	Acetone	11 ± 1	30 ± 2	8 ± 1
Stem	Water	0 ± 0	0 ± 0	0 ± 0
	Methanol	7 ± 0	18 ± 1	7 ± 0
	Ethanol	8 ± 1	19 ± 1	7 ± 0
	Acetone	8 ± 1	21 ± 1	7 ± 1
Tuber	Water	0 ± 0	0 ± 0	0 ± 0
	Methanol	0 ± 0	0 ± 0	0 ± 0
	Ethanol	0 ± 0	0 ± 0	0 ± 0
	Acetone	0 ± 0	9 ± 1	0 ± 0

## Data Availability

Not applicable.
