# Correction: Chen et al. Cytotoxic, Antibacterial, and Antioxidant Activities of the Leaf Extract of *Sinningia bullata*. *Plants* 2023, *12*, 859

**DOI:** 10.3390/plants12203557

**Published:** 2023-10-13

**Authors:** Pin-Jui Chen, En-Shyh Lin, Hsin-Hui Su, Cheng-Yang Huang

**Affiliations:** 1 Department of Biomedical Sciences, Chung Shan Medical University, Taichung City 402, Taiwan; 2 Department of Beauty Science, National Taichung University of Science and Technology, Taichung City 403, Taiwan; 3 Department of Pharmacy, Chia Nan University of Pharmacy and Science, Tainan City 717, Taiwan; 4 Department of Medical Research, Chung Shan Medical University Hospital, Taichung City 402, Taiwan

The authors would like to supplement some methodology information including the controls in the originally published version of this manuscript [1]. The ingredients of the leaf extract of *S. bullata* tentatively identified using GC-MS are also revised in this correction.

In “*4.2. Plant Materials and Extract Preparations*”, the following information is added as the first and second sentences: “*S. bullata* was collected south of Mount Babo Hagai (about an elevation of 400 m), Puli, Nantou County, Taiwan, in December 2020. This plant was identified by Dr. Zhong-Bao Zhang, the expert at Guoguang Flower Market and Taiwan Provincial Flower Marketing Cooperative, Taiwan.” The following information is also added as the last sentences: “Solvents (water, methanol, ethanol, and acetone) in extracts were removed using hot air circulation oven at 40 °C. Approximately 252 mg of Sb-T-M, 240 mg of Sb-S-M, 304 mg of Sb-L-M, 176 mg of Sb-T-E, 224 mg of Sb-S-E, 288 mg of Sb-L-E, 128 mg of Sb-T-A, 196 mg of Sb-S-A, 324 mg of Sb-L-A, 184 mg of Sb-T-W, 272 mg of Sb-S-W, and 290 mg of Sb-L-W were obtained from 1000 mg of dried plant powder. For all assays in this study, the extract powder was dissolved in 20% DMSO to make a stock solution at a concentration of 20 mg/mL. For the MTT and anticancer cell assays, the stock was diluted with the supplemented culture medium to the indicated assay concentrations. The cancer cells were incubated with the resulting extract solutions or the culture medium with 0.1% DMSO, referenced as the treatment or control group. For the antioxidant and antibacterial assays, the stock was diluted with 10% DMSO to the indicated assay concentrations.”

In “*4.3. Determination of TPC*”, the last sentence should read “GAE of different concentrations was used as the positive control, and the results of *S. bullata* extracts were compared with the standard curves of GAE and are expressed as mg equivalent/g dry weight of plant. Values show mean standard deviation of at least three independent experiments.” instead of “The results were compared with the standard curves of gallic acid (GAE) and were expressed as mg equivalent/g dry weight.”

In “*4.4. Determination of TFC*”, the last sentence should read “QUE of different concentrations was used as the positive control, and the results of *S. bullata* extracts were compared with the standard curves of QUE and are expressed as mg of QUE equivalent/g dry weight of plant. Values show mean standard deviation of at least three independent experiments.” instead of “The results are expressed as mg of quercetin (QUE) equivalent/g dry weight.”

In “*4.5. Determination of Antioxidant Activity by DPPH Radical Scavenging Assay*”, the following information is added after the first sentence and as the second, third, and fourth sentences: “DPPH was dissolved in absolute ethanol. Briefly, 180 μL of DPPH working solution was mixed with 20 μL of extracts in a 96-well plate and was incubated in the dark at RT for 15 min, and absorbance was measured using a microplate reader at 517 nm. L-Ascorbic acid dissolved in 10% DMSO was used as the positive control, with IC_50_ of 15.9 μg/mL. Values show mean standard deviation of at least three independent experiments.”

In “*4.6. GC-MS Analysis*”, the following information is added after the third sentence: “Helium was used as the carrier gas at a constant flow rate of 1 mL/min. The initial oven temperature was 40 °C and it was maintained at this temperature for 3 min; the temperature was gradually increased to 300 °C at a rate of 10 °C/min and was maintained for 1 min. The temperature of the injection port was 300 °C and the flow rate of helium was 1 mL/min. The compounds discharged from the column were detected using a quadrupole mass detector. The ions were generated using electron ionization method. The temperatures of the MS quadrupole and source were 150 and 300 °C, respectively; electron energy was 70 eV; the temperature of the detector was 300 °C; the emission current multiplier voltage was 1624 V; the interface temperature was 300 °C; and the mass range was from 29 to 650 amu.” instead of “[…] column were detected by a quadrupole mass detector. The ions were generated by the electron ionization method.”

In “*4.11. Antibacterial Activities*”, the following information is added as the last sentences: “Ampicillin and 10% DMSO were used as the positive and negative controls, respectively. For *E. coli*, *S. aureus,* and *P. aeruginosa*, the inhibition zones of ampicillin at concentration of 1 mg/mL were 14 ± 1, 26 ± 2, and 9 ± 1 mm, respectively. Values show mean standard deviation of at least three independent experiments.”

In “*4.13. MTT Cell Viability Assay*”, the following information is added as the last sentences: “EpoB (0, 1, 2, 5, 10, 15 nM) and the culture medium with 0.1% DMSO were used as the positive and negative controls, respectively. IC_50_ of EpoB was 7.54 nM. Values show mean standard deviation of at least three independent experiments.”

The authors noted that many diterpenoid compounds found in the leaf extract of *S. bullata* through GC–MS identification, described in the original published version of this manuscript, have never been found in other similar *Sinningia* species. In addition, some possible contaminants were also found in this extract. Thus, the authors would like to remove Table 5 from the original manuscript. The resulting revisions are as follows:

Table 5 is removed.

In the “Abstract”, the sentence “The top two contents in this extract were adlupulone and villosin.” is removed.

In “1. Introduction”, the last paragraph, the sentences “The most abundant two compounds in this extract were adlupulone and villosin. This might be why Sb-L-A possesses strong antibacterial and cytotoxic activities.” are removed.

In Figure 1C caption, the sentence “The most abundant two compounds in this extract were adlupulone and villosin.” is removed.

In “2.8. Gas Chromatography–Mass Spectrometry (GC–MS) Analysis of Sb-L-A”, this paragraph should read “[…] The GC chromatogram (Figure 1C) showed compounds detected in Sb-L-A. These compounds were identified by matching generated spectra with NIST 2011 and Wiley 10th edition mass spectral libraries. Contents tentatively identified in this extract were γ-tocopherol, β-sitosterol, stigmasterol, fuscumol, β-ionone, α-tocopherol, neophytadiene, 7,11-hexadecadienal, phytanol, stigmasta-3,5-diene, 2,4-di-tert-butylphenol, 3-(3,7-dimethyl-octa-2,6-dienyl)-4-hydroxy-benzaldehyde, octadecanoic acid, 3,7,11-trimethyl-1-dodecanol, cis-jasmonolactone, 6-[(2Z)-2-butenyl]-1,5,5-trimethyl-1-cyclohexene, δ-tocopherol, and phytol.”

In “*3. Discussion*”, the first paragraph, the last sentence “In addition, the active ingredients in Sb-L-A (Table 5) should be isolated and identified for further pharmacological applications.” is removed.

In “*3. Discussion*”, the second paragraph should read “[…] Accordingly, Sb-L-A was chosen for the GC–MS analysis to determine the active ingredients. Sb-L-A also had the highest TFC and cytotoxic and antibacterial activities. γ-Tocopherol, δ-tocopherol, and α-tocopherol were found in Sb-L-A. The antioxidant functions of these tocopherols (vitamin E) have been well confirmed in vivo and in vitro [37]. β-Ionone in Sb-L-A is also known to possess high antioxidant activities [38]. The antioxidation capacity of Sb-L-A might be from the co-effects of these compounds.”

In “3. Discussion”, the third paragraph should read “[…] could be the targets of the bioactive compounds of plants [39]. In this study, we examined the cytotoxic effects of Sb-L-A on the survival, apoptosis, proliferation, and migration of 4T1 (Figure 5) and B16F10 cells (Figure 4). Cell metastasis is a complicated process, which gradually leads to cancer propagation. The cancer cells are forced to lose epithelial-like features and invade the body through blood vessels, spreading into distant organs. In addition, this process is usually associated with drug resistance and disease recurrence [39]. Conventional cancer treatments commonly involve radiotherapy and chemotherapy but with several adverse effects and other critical disorders. Recently, natural products as potential anticancer agents have also been used and studied in many cancer models [35,40]. One significant advantage of using natural extracts against cancer cells is their multitargeted modes of action, which provide potential synergistic behavior and polypharmacology approaches for cancer therapies. Currently, promising plant-based anticancer medicines such as vincristine, vinblastine, and paclitaxel have been developed and used in clinical applications [4,5]. β-Ionone identified in Sb-L-A is also known to have significant antiproliferative, antimetastatic, and apoptosis-induction activities, both in vitro and in vivo [41]. Given that the combination of Sb-L-A (20 μg/mL) with the anticancer drug EpoB (2 nM) could synergistically enhance the cytotoxicity against the B16F10 cells (Figure 7), how Sb-L-A can co-act with EpoB to improve the chemosensitivity of EpoB should be further elucidated.”

In “3. Discussion”, the fourth paragraph should read “Cancer progression is associated with the dysfunction of checkpoint controls, which regulate normal passage through the cell cycle [42]. The G2 cell-cycle checkpoint is a critical genome guardian of tumor cells and, therefore, G2 checkpoint abrogation has been considered to be a promising therapeutic anticancer target, such as several cell cycle proteins [42]. The flow cytometry results indicated that Sb-L-A could promote the distribution of the G2 phase and decreased the cell proportion in the G1 and S phases in B16F10 melanoma cells (Figure 6). Accordingly, Sb-L-A might suppress melanoma cell proliferation by inducing G2 cell-cycle arrest. Currently, we are investigating the cellular signaling pathways that trigger this G2 arrest in B16F10 melanoma cells.”

In “3. Discussion”, the fifth paragraph, these sentences “Through the GC–MS analysis (Table 5), the top content in Sb-L-A was adlupulone (28.88%). Adlupulone [51] and lupulone [52] are also abundant in hop extracts and may mainly act to fight infections by pathogens [53]. These compounds might be useful alone or in combination with other active ingredients in Sb-L-A for antibacterial function. Further research should focus on determining the molecular target(s) and the antibacterial mechanism of adlupulone and lupulone.” are removed.

In “3. Discussion”, a new paragraph is added as the sixth paragraph: “Besides *S. bullata*, some other Sinningia species also have cytotoxic, antibacterial, and antioxidant activities. There are two scientific articles, clearly indicating that extracts of *S. allagophylla* [43] and *S. mauroana* [44] have strong cytotoxic activities. Compounds isolated from *S. aggregata* [45], *S. reitzii* [46], and *S. leucotricha* [47] have been further analyzed in cytotoxic activities. The antibacterial and antioxidant properties of extracts and the isolated compounds from *S. hatschbachii* [48], *S. magnifica* [49], *S. aggregata* [50], *S. warmingii* [51], *S. mauroana* [44,52], and *S. reitzii* [53] have also been investigated experimentally. Unlike results in this study, however, most of these extracts did not have significant antibacterial activities. Whether these significant disparities are due to inherent differences among the species, different growth conditions of the plants (the soil, fertilizer, altitude, and so on), additional plant–environment interactions, the use of different assay methods, the use of different solvent for extractions, and/or the effect of different investigators remains unknown. We noticed that the focusing studied targets of these Sinningia plants are different. Although we prepared different extracts from various parts of *S. bullata*, i.e., the leaf, stem, and tuber, we only found that the leaf extracts obtained using acetone had the highest cytotoxic activities, antioxidation capacity, and antibacterial activity against the Gram-positive *S. aureus*. Similarly, as in most studies mentioned above, tuber extracts of *S. bullata* did not show any antibacterial activity (with the inhibition zone of 0) (Table 4). Furthermore, even using acetone as the extraction solvent, tuber extracts of *S. bullata* also had very low cytotoxic activities as compared to the leaf extract (Figure 3). Accordingly, the most abundant compounds in this extract were tentatively deduced using GC–MS. We also noted that some compounds contained in tubers of Sinningia species, mainly analyzed by using nuclear magnetic resonance spectroscopy, such as aggregatin E [45], aggregatin F [45], leucotrichoic acid [47], 6-hydroxy-7-methoxy-2-O-methylduniol [48], 5,6-dihydroxy-7-methoxy-α-dunnione [53], 8-hydroxy-6,7-dimethoxy-α-dunnione [53], warmingiin A [51], and warmingiin B [51], were not found in our study. Although GC–MS is widely used in the analysis of chemical compositions in plant extract and provides enhanced sample identification, higher sensitivity, and an increased range of analyzable samples in a reasonable time, GC–MS is not capable of directly analyzing compounds in plant extract that are nonvolatile, polar, or thermally labile. It is possible that the major compounds in the acetone extract of *S. bullata* leaf have not been detected via GC–MS. Given that some plant medicines are only produced in some specific species, for example, for artemisinin that is abundant in *Artemisia annua*, but not in *Artemisia apiacea*, more studies should be needed to determine the chemical compositions of different parts of these different *Sinningia* plants for further possible medical applications. To understand the cytotoxic mechanisms of the acetone extract, our laboratory is currently attempting to determine the molecular target(s) in the Gram-positive *S. aureus* and the melanoma cell.”

In “References”, the following references are also revised:

38. Asokkumar, S.; Naveenkumar, C.; Raghunandhakumar, S.; Kamaraj, S.; Anandakumar, P.; Jagan, S.; Devaki, T. Antiproliferative and antioxidant potential of beta-ionone against benzo(a)pyrene-induced lung carcinogenesis in Swiss albino mice. *Mol. Cell. Biochem.* **2012**, *363*, 335–345.
39. Pastushenko, I.; Blanpain, C. EMT Transition States during Tumor Progression and Metastasis. *Trends Cell Biol.* **2019**, *29*, 212–226.
40. Bailly, C. Yuanhuacin and Related Anti-Inflammatory and Anticancer Daphnane Diterpenes from Genkwa Flos-An Overview. *Biomolecules* **2022**, *12*, 192.
41. Ansari, M.; Emami, S. β-Ionone and its analogs as promising anticancer agents. *Eur. J. Med. Chem.* **2016**, *123*, 141–154.
42. Otto, T.; Sicinski, P. Cell cycle proteins as promising targets in cancer therapy. *Nat. Rev. Cancer* **2017**, *17*, 93–115.
43. Riva, D.; Barison, A.; Stefanello, M.É.A.; Poliquesi, C.B.; Ruiz, A.L.T.G.; Salvador, M.J. Estudo químico de *Sinningia allagophylla* guiado por testes de atividade antiproliferativa. *Quim. Nova* **2012**, *35*, 974–977.
44. Winiewski, V.; Serain, A.F.; Sá, E.L.; Salvador, M.J.; Stefanello, M.É.A. Chemical constituents of *Sinningia mauroana* and screening of its extracts for antimicrobial, antioxidant and cytotoxic activities. *Quim. Nova* **2020**, *43*, 181–187.
45. Verdan, M.H.; Mera de Souza, L.; Ernesto de Carvalho, J.; Vendramini Costa, D.B.; Salvador, M.J.; Barison, A.; Alves Stefanello, M.É. Two new hydronaphthoquinones from *Sinningia aggregata* (Gesneriaceae) and cytotoxic activity of aggregatin D. *Chem. Biodiv*. **2015**, *12*, 148–152.
46. Silva, A.S.; Amorim, M.S.; Fonseca, M.M.; Salvador, M.J.; Sá, E.L.; Stefanello, M.É.A. A new cytotoxic naphthoquinone and other chemical constituents of *Sinningia reitzii*. *J. Braz. Chem. Soc.* **2019**, *30*, 2060–2065.
47. Verdan, M.H.; Unemoto, L.K.; Faria, R.T.; Salvador, M.J.; Sá, E.L.; Barison, A.; Stefanello, M.É.A. Leucotrichoic acid, a novel sesquiterpene from *Sinningia leucotricha* (Gesneriaceae). *Tet. Let*. **2013**, *54*, 4735–4737.
48. Amorim, M.S.; Serain, A.F.; Salvador, M.J.; Stefanello, M.É.A. Chemical constituents of *Sinningia hatschbachii*. *Nat. Prod. Commun.* **2017**, *12*, 1763–1764.
49. Serain, A.F.; Silvério, S.E.B.; De Lourenço, C.C.; Nunes, V.K.; Corrêa, W.R.; Stefanello, M.É.A.; Salvador, M.J. Development of *Sinningia magnifica* (Otto & A. Dietr.) Wiehler (Gesneriaceae) tissue culture for in vitro production of quinones and bioactive molecules. *Ind. Crops Prod.* **2021**, *159*, 113046.
50. Verdan, M.H.; Barison, A.; Sá, E.L.; Salvador, M.J.; Poliquesi, C.B.; Eberlin, M.N.; Stefanello, M.É.A. Lactones and quinones from the tubers of *Sinningia aggregata*. *J. Nat. Prod.* **2010**, *73*, 1434–1437.
51. Winiewski, V.; Verdan, M.H.; Ribeiro, M.A.; Hernandez-Tasco, A.J.; Salvador, M.J.; Stefanello, M.É.A. Warmingiins A and B, two new dimeric naphthoquinone derivatives from *Sinningia warmingii* (Gesneriaceae). *J. Braz. Chem. Soc.* **2017**, *28*, 598–602.
52. Winiewski, V.; Fonseca, M.M.; Salvador, M.J.; Sá, E.L.; Stefanello, M.É.A. Three new naphthoquinones from the tubers of *Sinningia mauroana*. *Nat. Prod. Res.* **2023**, *37*, 263–268.
53. Winiewski, V.; Silva, A.S.; Alvarez, K.D.C.; Sá, E.L.; Salvador, M.J.; Stefanello, M.É.A. Antioxidant naphthoquinones of *Sinningia reitzii* from Santa Catarina State, Brazil. *Quim. Nova* **2021**, *44*, 284–287.

The authors apologize for any inconvenience caused and state that the conclusion that the leaf extract of *Sinningia bullata* has cytotoxic, antibacterial, and antioxidant activities is still unaffected. The original publication has also been updated.
